# Genome-Wide Analysis of SQUAMOSA-Promoter-Binding Protein-like Family in Flowering *Pleioblastus pygmaeus*

**DOI:** 10.3390/ijms232214035

**Published:** 2022-11-14

**Authors:** Wenjing Yao, Chuanzhe Li, Huajun Fu, Meng Yang, Hongyu Wu, Yulong Ding, Long Li, Shuyan Lin

**Affiliations:** 1 Co-Innovation Center for Sustainable Forestry in Southern China/Bamboo Research Institute, Nanjing Forestry University, 159 Longpan Road, Nanjing 210037, China; 2 Huaiyin Institute of Agricultural Sciences of Xuhuai Region, Jiangsu Academy of Agricultural Sciences, Huaian 223001, China

**Keywords:** *Pleioblastus pygmaeus*, SPLs, reproductive growth, flora transition, flower organ development, *miRNA156*

## Abstract

SQUAMOSA Promoter-Binding Protein-Like (SPL) family is well-known for playing an important role in plant growth and development, specifically in the reproductive process. Bamboo plants have special reproductive characteristics with a prolonged vegetative phase and uncertain flowering time. However, the underlying functions of SPL genes in reproductive growth are undisclosed in bamboo plants. In the study, a total of 28 SPLs were screened from an ornamental dwarf bamboo species, *Pleioblastus pygmaeus*. Phylogenetic analysis indicates that 183 SPLs from eight plant species can be classified into nine subfamilies, and the 28 *PpSPLs* are distributed among eight subfamilies. Homologous analysis shows that as many as 32 pairs of homologous genes were found between *P*. *pygmaeus* and rice, and 83 pairs were found between *P*. *pygmaeus* and Moso bamboo, whose Ka/Ks values are all <1. MiRNA target prediction reveals that 13 out of the 28 *PpSPLs* have recognition sites complementary to *miRNA156*. To screen the SPLs involved in the reproductive growth of bamboo plants, the mRNA abundance of the 28 *PpSPLs* was profiled in the different tissues of flowering *P*. *pygmaeus* and non-flowering plants by RNA-Seq. Moreover, the relative expression level of eight *PpSPLs* is significantly higher in flowering *P*. *pygmaeus* than that in non-flowering plants, which was also validated by RT-qPCR. Combined with phylogenetic analysis and homologous analysis, the eight significant, differentially expressed *PpSPLs* were identified to be associated with the reproductive process and flower organ development. Among them, there are four potential *miRNA156*-targeting *PpSPLs* involved in the flowering process. Of significant interest in the study is the identification of 28 SPLs and the exploration of four key flowering-related SPLs from *P*. *pygmaeus*, which provides a theoretic basis for revealing the underlying functions of SPLs in the reproductive growth of bamboo plants.

## 1. Introduction

As one of the most potential and most efficient renewable evergreen species for bioenergy and carbon fixation, bamboo has the advantages of rapid growth, a large biomass, and wide use [1,2]. Bamboo plants also have special reproductive habits with unpredictable, gregarious, and uncontrollable flowering [3]. Different bamboo species display a wide variation in blooming style and flowering time [3,4]. In particular, large-scale death after gregarious blossoming leads to the decline of bamboo forests, which limits the resource utilization of many bamboo species, and may cause huge economic losses and induce threats to biodiversity [4,5]. The special reproductive characteristics and genetic complexity retard the exploration of the flowering mechanism of bamboo plants when compared to other *Poaceae* plants, such as rice. Therefore, extensive research is essential to reveal the flowering mechanism of bamboo plants, which contributes to their cultivation and conservation.

Transcription factors (TFs) are characterized by the fact that they contain one or more DNA-binding domains (DBDs) through which they bind to specific promoter sequences to enhance or block the expression of their downstream target genes [6,7]. A gene family is a set of several similar genes generally with a similar structure and function, formed by the duplication of a single original gene. It comes from whole genome duplication or polyploidization, segmental duplication, tandem duplication, or replicative transposition in the long evolutionary process [8,9,10]. Belonging to one of the most ubiquitous plant-specific TF families, SQUAMOSA Promoter-Binding Protein-Like (SPL) family members are heterogeneous in primary structure and share a highly conserved DBD containing 10 conserved cysteine and histidine residues. Moreover, there is a conservative nuclear localization signal (NLS) at C-terminal and two zinc finger structures at N-terminal in the conserved domain [11,12]. SPL family genes originate from *Antirrhinum majus* and have been successively identified from many plant species, such as *Arabidopsis thaliana* (17 members), *Physcomitrella patens* (13), *Zea mays* (31)*, Oryza sativa* (19), *Ananas comosus* (15)*, Brachypodium distachyon* (17)*, Sorghum bicolor* (19), *Phyllostachys edulis* (32), etc. [13,14,15,16,17,18,19,20,21].

SPL genes mainly play essential roles in plant growth and development, specifically in reproductive growth [12,14]. An increasing number of reports have proved their positive functions in flora transition and the subsequent development of flower organs [22,23,24,25,26]. For example, *SPL2* was proved to control reproduction process and flower organ development by activating ASYMMETRIC LEAVES 2 (*AS2*) in *Arabidopsis* [23]. *OsSPL16* promotes cell division and grain filling, which has positive consequences for grain width and the yield of rice [24]. In addition, some SPL genes have specific functions on reproductive growth by affecting signal transduction and stress resistance indirectly. For instance, *SPL8* and *miR156*-targeted SPL genes redundantly regulate gynoecium differential patterning by interfering with auxin homeostasis and signal transduction in *Arabidopsis* [25]. Overexpression of *SPL1* or *SPL12* can improve the heat tolerance of *Arabidopsis* and *tobacco* during the reproduction process [26]. The most widespread reported function of SPL genes is to promote the juvenile to adult transition, which induces floral inductive signals and results in flowering [27,28]. As an example, *Arabidopsis SPL3, SPL4,* and *SPL5* can enhance the FLOWERING LOCUS T (*FT*) *-FD* module by targeting a few flowering regulators such as *AP1, LFY,* and *FUL* in the flowering pathway [29,30]. In particular, *miRNA156*-*SPLs* act as a regulatory hub in the age pathway, which controls the flowering time and phase transition in *Arabidopsis* [28,31]. For example, *miRNA156*-*SPL3* prevents early flowering by translational inhibition in *Arabidopsis* [32]. In addition, *miRNA156* and its target SPLs also participate in vegetative growth. For instance, *miR156*-*SPL10* was proved to modulate lateral root development, branching, and leaf morphology in *Arabidopsis* [33].

*P. pygmaeus* is a kind of ornamental dwarf bamboo species with a high ecological and economic value in China [34,35]. During the years of 2015–2018, *P. pygmaeus* flowered in the bamboo garden of Nanjing Forestry University, which provided a great opportunity for validating the underlying functions of SPL genes in bamboo plants [34,35]. In this study, we identified a total of 28 SPL family members from the transcriptome of *P. pygmaeus*, and 13 of them were predicted to be potential *miRNA156*-targeting *PpSPLs*. In addition, we profiled the expression pattern of the 28 *PpSPLs* in the different tissues of flowering *P. pygmaeus* and non-flowering plants by RNA-Seq. A total of eight *PpSPLs* were found to be highly expressed in flowering *P*. *pygmaeus,* which was also validated by RT-qPCR. Moreover, we conducted phylogenetic analysis and homologous analysis of the 183 SPL genes from *P. pygmaeus* and seven other plant species. Combined with theoretic analysis, the eight significant, differentially expressed *PpSPLs* were identified to participate in reproductive growth and flower organ development, and four of them are potential *miRNA156* targets involved in the flowering process, which is our research emphasis in the future.

## 2. Results

### 2.1. Identification and Sequence Analysis of SPL Genes from Pleioblastus pygmaeus

**Figure 1:**
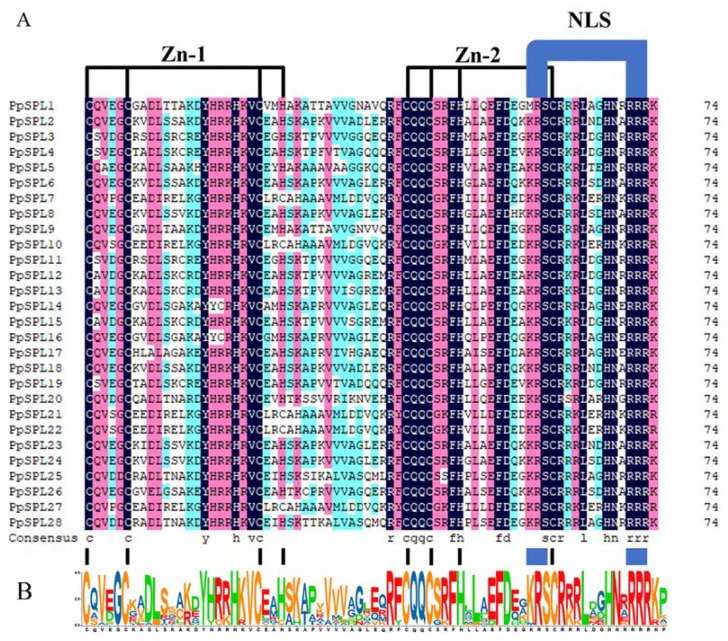
Alignment of SBP domains in *PpSPLs*. (**A**). Multiple alignment of conserved SBP domain, which contains two conserved zinc finger structures (Zn-1, Zn-2) and a nuclear localization signal (NLS). (**B**). The sequence markers of conserved SBP domain in *PpSPLs*.

In the study, a total of 28 SPL genes were identified from *P. pygmaeus* and named as *PpSPL1-28*. The conserved domain of the *PpSPLs* contains 75 amino acid residues, including two zinc finger motifs and a highly conserved NLS (Figure 1). The protein sequences of *PpSPLs* are 154–1117 aa with a molecular weight of 17,009.18–122,036.59 Da and an isoelectric point of 5.35–10.83. The fat index and unstable index of *PpSPLs* are 47.6–81.7 and 49.27–67.67, respectively. Based on hydrophilic index, most *PpSBPs* belong to hydrophilic proteins, among which *PpSPL24* is the strongest (−0.659) and *PpSPL1* is the weakest (−0.268). Subcellular localization prediction shows that all 28 *PpSPLs* are more likely to exist in the nucleus (Supplemental Table S1).

### 2.2. Phylogenetic Analysis of SPL Family Genes

**Figure 2:**
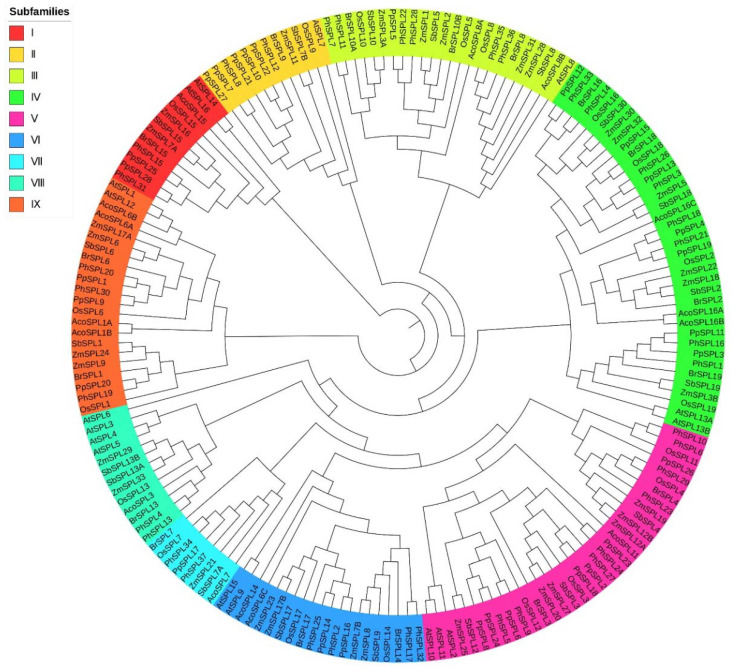
Phylogenetic tree of 28 *PpSPLs* and 155 SPLs from 7 other plant species. The phylogenetic tree of the 183 SPL genes was constructed using the ML (maximum likelihood) method by RAxML. The tree can be divided into nine groups and each color represents a group. *AtSPLs*, the SPLs from *Arabidopsis thaliana*; *AcoSLPs*, the SPLs from *Ananas comosus*; *BrSPLs*, the SPLs from *Brachypodium distachyon*; *OsSLPs*, the SPLs from *Oryza sativa*; *PhSLPs*, the SPLs from *Phyllostachys edulis*; *PpSLPs*, the SPLs from *Pleioblastus pygmaeus*; *SbSLPs*, the SPLs from *Sorghum bicolor*; *ZmSLPs*, the SPLs from *Zea mays*.

A phylogenetic tree of 183 SPL genes from *P. pygmaeus* and seven other plant species was constructed using the ML method, which can be classified into nine subfamilies (Figure 2). The subfamilies, I, III, IV, V, VI, and IX, included the SPL members from all eight plant species. The 28 *PpSPLs* are distributed in the eight subfamilies except for VIII. Among them, the largest subgroup is the IV clade with 38 SPLs, including seven *PpSPLs*; followed by the V with 34 SPLs, including seven *PpSPLs*; and the smallest subgroup is the VII with eight members, including 1 *PpSPL*. *A. thaliana* has the most SPL members in the subfamily VIII, while the other seven plant species have the most members in the subfamily IV or V.

### 2.3. Homologous Analysis of SPL Family Genes

A total of 5, 2, 3, 17, 2, 3, 39, and 34 paralogous gene pairs were found in *A. thaliana*, *A. comosus*, *S. bicolor*, *Z. mays*, *O. sativa*, *B*. *distachyon*, *Ph. edulis*, and *P. pygmaeus*, respectively. Nineteen *OsSPLs*, 32 *PhSPLs*, and 28 *PpSPLs* were used for the identification of orthologous genes. There are 32 pairs of orthologous genes between *P. pygmaeus* and rice, 83 between *P. pygmaeus* and *Ph. edulis*, and 46 between rice and *Ph. edulis* (Supplemental Table S2). The ratio of the number of nonsynonymous substitutions per nonsynonymous site (Ka) to the number of synonymous substitutions per synonymous site (Ks) of the above orthologous pairs are all <1, which indicates that the SPLs retain a conservative sequence and a similar function after purification and selection in the three monocotyledons. In addition, the number of orthologous gene pairs between *Ph. edulis* and *P. pygmaeus* is significantly more than that in the other two pairs, which may be related to the obvious difference of flowering habits between bamboo plants and other Gramineae plants.

### 2.4. Motif Analysis of SPL Family Genes

**Figure 3:**
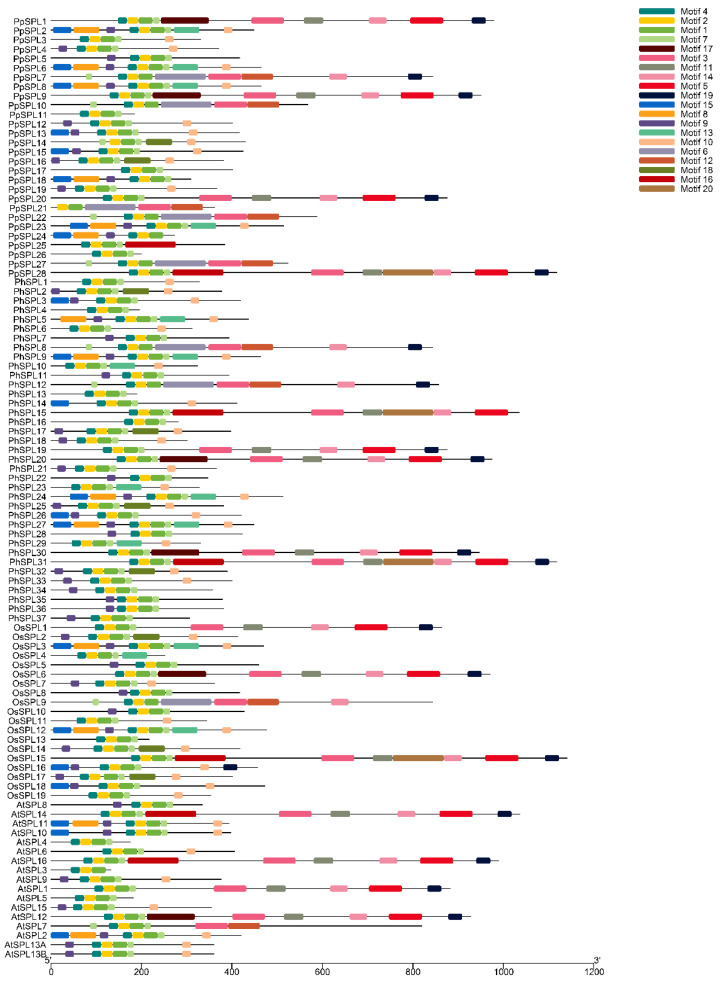
Motif distribution of SPL genes from *Arabidopsis thaliana*, *Oryza sativa*, *Phyllostachys edulis*, and *Pleioblastus pygmaeus*. *AtSPLs*, the SPLs from *Arabidopsis thaliana*; *OsSLPs*, the SPLs from *Oryza sativa*; *PhSLPs*, the SPLs from *Phyllostachys edulis*; *PpSLPs*, the SPLs from *Pleioblastus pygmaeus*.

The SPL members from *A. thaliana*, *O. sativa*, *Ph. edulis*, and *P. pygmaeus* were used for motif analysis. A total of 20 different motifs were found in the SPL genes from the four plant species (Figure 3). Of those, Motif 1, Motif 2, and Motif 4 are directly adjacent and shared in all SPL genes, which indicates that they are related to domain conservation. As expected, there are more homologous genes among the plant species with a closer genetic relationship, which also share more common motif sequences. According to sequence length, SPL family genes can be divided into two types, including the short SPLs with a length of 400 ± 200 bp and the long ones with a length of 900 ± 200 bp. There are more than 10 motifs distributed in the long SPLs, such as *PhSPL31*, *AtSPL12*, *PpSPL28*, and *OsSPL15*, whereas only three shared motifs exist in the short ones, such as *AtSPL3*. In particular, many SPLs contain the exact same motifs, such as *PpSPL2*, *PpSPL6, PpSPL8*, *PhSPL9*, *PhSPL27*, *OsSPL3*, and *OsSPL12*, which also implies their function similarity.

### 2.5. miRNA Target Analysis

**Figure 4:**
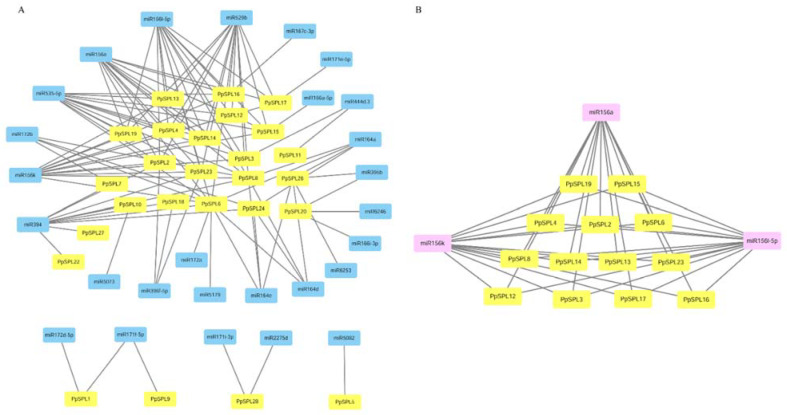
miRNA target analysis of 28 *PpSPLs*. (**A**) The relationship of 28 *PpSPLs* with all kinds of miRNAs. (**B**) The *miRNA156*-targeting *PpSPLs*. The yellow icons represent *PpSPLs*, the blue icons represent *miRNAs*, and the pink icon represents *miRNA156*.

Except for *PpSPL21* and *PpSPL25*, the other 26 *PpSPLs* were predicted to have a likely target relationship with *miRNAs*, mainly including *miR156*, *miR164*, *miR172*, *miR394*, *miR529*, and *miR535* (Figure 4A, Supplemental Excel S1). Among them, *miRNA535* were predicted to have the most abundant targeting SPLs, which contains 14 *PpSPLs*, including *PpSPL2*/*3*/*4*/*6*/*8*/*12*/*13*/*14*/*15*/*16*/*19*/*20*/*23*/*26*. *miR156* and *miR529* followed with 13 of the same targeting *PpSPLs*, including *PpSPL2*/*3*/*4*/*6*/*8*/*12*/*13*/*14*/*15*/*16*/*17*/*19*/*23*. *miR394* was predicted to target *PpSPL2*/*6*/*7*/*8*/*10*/*18*/*22*/*23*/*24*/*27*, *miR172* to target *PpSPL1*/*6*/*7*/*8*/*24*, and *miR164* to target *PpSPL6*/*8*/*24*/*26*. In particular, we screened out the 13 target *PpSPLs* of *miRNA156* and focused on the expression pattern analysis of the SPLs (Figure 4B, Supplemental Excel S1).

### 2.6. Expression Pattern Analysis of PpSPL Genes

**Figure 5:**
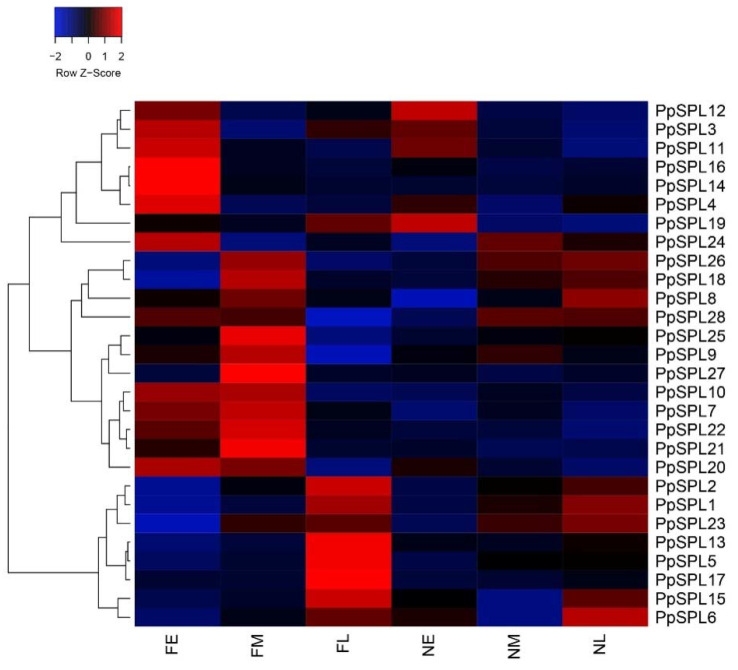
Expression pattern of *PpSPLs* in the different tissues of flowering *Pleioblastus pygmaeus* and non-flowering plants. FE, dormant shoot buds from flowering *P. pygmaeus*; FM, germinated shoots from flowering *P. pygmaeus*; FL, flower buds from flowering *P. pygmaeus*; NE, dormant shoot buds from non-flowering *P. pygmaeus*; NM, germinated shoots from non-flowering *P. pygmaeus*; NL, leaf buds from non-flowering *P. pygmaeus*.

We profiled the expression pattern of 28 *PpSPLs* in the different tissues of flowering and non-flowering *P. pygmaeus* based on RNA-Seq. As shown in Figure 5, except for *PpSPL12*, *PpSPL19*, and *PpSPL6*, the relative expression level of most *PpSPLs* was higher in the flowering *P. pygmaeus* than that in the non-flowering plants. According to the expression pattern in flowering *P. pygmaeus*, the *PpSPLs* can be generally classified into three types. The first type contains six *PpSPLs*, including *PpSPL3*, *PpSPL11*, *PpSPL16*, *PpSPL14*, *PpSPL4*, and *PpSPL24*, which are highly expressed in the dormant shoot buds (FE). The 12 SPLs, including *PpSPL26*, *PpSPL18*, *PpSPL8*, *PpSPL28*, *PpSPL25*, *PpSPL9*, *PpSPL27*, *PpSPL10*, *PpSPL7*, *PpSPL22*, *PpSPL21*, and *PpSPL20* belong to the second type, which are highly expressed in the germinated shoots (FM). The third type consists of seven *PpSPLs,* including *PpSPL2*, *PpSPL1*, *PpSPL23*, *PpSPL13*, *PpSPL5*, *PpSPL17*, and *PpSPL15*, which are highly expressed in the flower buds (FL). Among them, the relative expression level of eight *PpSPLs* in flowering *P. pygmaeus* was significantly higher than that in non-flowering plants, including two highly expressed genes in FE (*PpSPL14* and *PpSPL16*), three in FM (*PpSPL21*, *PpSPL25*, and *PpSPL27*), and three in FL (*PpSPL5*, *PpSPL13*, and *PpSPL17*).

**Figure 6:**
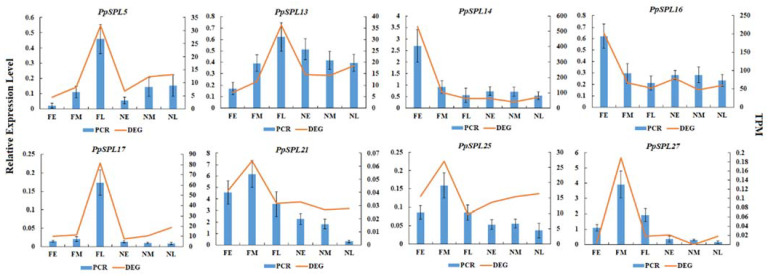
RT-qPCR validation of significant, differentially expressed *PpSPLs* in *Pleioblastus pygmaeus*. FE, dormant shoot buds from flowering *P. pygmaeus*; FM, germinated shoots from flowering *P. pygmaeus*; FL, flower buds from flowering *P. pygmaeus*; NE, dormant shoot buds from non-flowering *P. pygmaeus*; NM, germinated shoots from non-flowering *P. pygmaeus*; NL, leaf buds from non-flowering *P. pygmaeus*; PCR, relative expression level by RT-qPCR; DEG, differentially expressed gene by RNA-Seq; TPM, Transcripts Perkilobase Million. Red lines represent the mRNA abundance of SPLs by RNA-Seq analysis. Blue bars represent the relative expression level of SPLs by RT-qPCR. Mean values and deviations were calculated from three independent biological experiments.

To validate the expression pattern of the eight significant, differently expressed *PpSPLs* in *P. pygmaeus*, their relative expression level in different tissues was further confirmed by RT-qPCR. As shown in Figure 6, the expression trends of the eight *PpSPLs*, quantified by RT-qPCR, are generally consistent with RNA-Seq.

## 3. Discussion

As a major plant-specific TF family, the SPL family plays an important role in plant growth and development. In particular, many SPL genes are well-known for regulating the plant reproductive process, such as vegetative phase change, flora transition, and flowering time [14,28,31]. Due to special reproductive characteristics and sampling difficulty, the flowering mechanism is unrevealing in bamboo plants, as well as the biological functions of SPLs in reproductive growth. In the study, we identified a total of 28 SPL family members from *P. pygmaeus* and profiled their expression pattern in the different tissues of flowering *P. pygmaeus* and non-flowering plants. We also conducted phylogenetic analysis and homologous analysis of the 28 *PpSPLs*, which provides a functional and comprehensive reference of the single SPL gene in bamboo plants.

Phylogenetic analysis is of great significance for family evolution and functional divergence, which provides important references for the characterization of unknown genes [36,37]. To identify the SPLs from *P*. *pygmaeus*, whose genome information was lacking up to now, a phylogenetic tree of 28 *PpSPLs* and 155 SPLs from seven other plant species was constructed in the study. Here, we focus on the phylogenetic analysis of the SPLs between *P*. *pygmaeus* and the model plant *Arabidopsis*. There is a total of 19 SPLs from *Arabidopsis*, which are distributed in the eight subfamilies, except for VII. The 28 *PpSPLs* are classified into the eight clades, except for VIII. However, *Arabidopsis* has the most SPLs in the clade-VIII, including *AtSPL3*–*6*, which are post-transcriptionally regulated by *miRNA156* [14,38]. Among them, *AtSPL6* has a positive function in regulating defense genes in innate immunity [39]. *AtSPL3*–*5* were proved to promote reproductive transition by regulating a few flowering regulators, such as *AP1, LFY,* and *FUL* [29,30]. The fact that subfamily VIII has no orthologous *PpSPLs* indicates functional evolution following speciation and gene duplication in this clade. The largest subfamily, clade-IV, occurs three times of gene duplication events that *P*. *pygmaeus* all participates in, contributing to the most *PpSPL* members, including *PpSPL3*, *4*, *11*, *12*, *13*, *15*, and *19*. *Arabidopsis* has only two identified SPLs in this clade, *AtSPL13A* and *AtSPL13B*, which have been implicated in the regulation of post-germinative transition from the cotyledon to vegetative-leaf stages [40]. It is inferred that the above seven *PpSPLs* may have functions in phrase transition. Similar to clade-IV, clade-V also has seven *PpSPLs*, including *PpSPL2*, *6*, *8*, *18*, *23*, *24*, and *26*, which occurs two times of gene duplication events. There are three orthologous *AtSPLs* in this clade, including *AtSPL2*, *AtSPL10*, and *AtSPL11*, which were reported to control plant morphology, plant reproduction, and flower organ development by targeting related genes [12,23,33]. Functional analysis within clade-II is limited to *AtSPL7* and supports the role of *PpSPL7*/*10*/*21*/*22*/*27* in regulating copper homeostasis [41]. *AtSPL14* and *AtSPL16* exist for the function annotation of *PpSPL25*/*28* in the clade-I. *AtSPL14* was proved to delay the transition from juvenile to adult and improve fumonisin sensitivity, while *AtSPL16* is a non-functional duplication of *AtSPL14* [42,43]. There is a pair of homologous pairs in the subfamily III, *PpSPL5* and *AtSPL8*. *AtSPL8* has a positive effect on pollen sac development and seed set, which is also reported in *AtSPL9* and *AtSPL15* [22,44]. However, *AtSPL9* and *AtSPL15* are classified into subfamily VI, which indicates either functional conservation or independent recruitment of SPL genes. The two *AtSPLs* were also reported to regulate shoot maturation, leaf initiation, and flowering time by miRNA: target gene interactions [45,46]. This indicates that *PpSPL14*/*16* in the same group may have similar diverse functions with *AtSPL9* and *AtSPL15*. In the subfamily IX, *PpSPL1*, *PpSPL9*, and *PpSPL20* are classified with *AtSPL1* and *AtSPL12*, which are involved in plant thermotolerance at the reproductive stage [26]. In summary, based on phylogenetic analysis, there are as many as 19 *PpSPLs* involved in phrase transition (*PpSPL3*/*4*/*11*/*12*/*13*/*15*/*19*/*25*/*28*), flowering (*PpSPL14*/*16*), and flower organ development (*PpSPL2*/*5*/*6*/*8*/*14*/*16*/*18*/*23*/*24*/*26*). Combined with transcriptome screening of the eight significant, differentially expressed *PpSPLs*, including *PpSPL5*/*13*/*14*/*16*/*17*/*21*/*25*/*27*, *PpSPL5*/*14*/*16* are likely to be associated with flower organ development, *PpSPL13*/*25* may be involved in phrase transition, and *PpSPL14*/*16* have the potential to participate in flowering.

The pangenomic era has witnessed a great advance in clade-specific homology inference with increasing genome sequencing of plant species. A few plant species can even have multiple high-quality references [47]. Due to genetic complexity, the genome information of most bamboo plants is not easily accessible. Homologous analysis becomes a soft option for prior assessments of unknown genes in bamboo plants. Compared to *Zea mays* and triticeae crops, the SPLs from bamboo plants have similar sequence features with those from rice, such as GC content and codon usage. This indicates that bamboo is a close relative of rice, which is also reflected from phylogenetic analysis and motif composition. To explore the biological functions of SPLs from *P. pygmaeus*, we focused on the homologous analysis of *OsSPLs* and *PpSPLs*. In the study, as many as 32 pairs of orthologous genes were found between rice and *P. pygmaeus*. Specifically, there are five homologous pairs including *OsSPL3*/*12*, *OsSPL4*/*11*, *OsSPL5*/*10*, *OsSPL14*/*17*, and *OsSPL16*/*18* involved in the reproduction growth in *Oryza sativa* [48]. *OsSPL16*/*18* are positive regulators of cell proliferation and contribute to the grain weight and yield of rice, indicating the two genes function in floral organs and seeds [24,49]. As orthologous genes of *OsSPL16*/*18*, *PpSPL13*/*15* are highly expressed in FL. According to the studies of Yang et al. [50] and Shao et al. [51], *OsSPL5*/*10* have similar functions to *OsSPL16*/*18*, although they are classified into a different clade than *OsSPL16*/*18*. *PpSPL5* is the orthologous genes of *OsSPL5*/*10*, which also indicates its role in tiller number, setting rate, heading date, and germination rate [52]. Interestingly, the relative expression level of *PpSPL5* is significantly higher in FL than in NL. *PpSPL17* is also highly expressed in FL, and its homologous gene, *OsSPL7*, was reported to regulate the tiller number, panicle architecture, and grain size [53,54]. The above indicates that *PpSPL5*/*13*/*15*/*17* may function in flowering and/or flower organ development. *OsSPL14* was well-known for defining plant architecture and promote panicle branching and grain productivity in rice [55,56]. Its sister paired gene, *OsSPL17*, plays a role in panicle size, plant architecture, and root development [52,54]. *PpSPL14*/*16* have a close homologous relationship with *OsSPL14*/*17*. The phenomenon that new shoots of *P. pygmaeus* bloom in a flowering forest indicates that flora transition has been activated at shoot bud stage in bamboo plants [35]. In particular, the two *PpSPLs* are highly expressed in FE, which implies that *PpSPL14*/*16* participate in reproductive processes. The homologous pairs *OsSPL3* and *OsSPL12* are highly expressed in the leaves and panicles, respectively, which regulate heading date and panicle size, respectively [52]. This fact reflects that the different expression pattern of pair genes may contribute to an alternative explanation of sub-functionalization and neofunctionalization. As respective homologous genes of *OsSPL3* and *OsSPL12*, *PpSPL2*/*18*/*23* and *PpSPL6*/*8* adopt different spatial expression patterns with the two *OsSPLs*. The expression of the five *PpSPLs* displays no significant difference between flowering *P. pygmaeus* and non-flowering plants. *OsSPL4* and *OsSPL11* are a pair of homologous genes in rice. *OsSPL4* affects heading date, grain size, and grain yield, while *OsSPL11* affects setting rate and tiller number [52,57]. *PpSPL26* is a homologous gene of *OsSPL4*, which is highly expressed in FM, indicating its role in flowering and reproductive growth. Based on RNA-Seq and RT-qPCR, the relative expression level of *PpSPL21/25/27* is highly expressed in FM. Among them, *PpSPL21* and *PpSPL27* are both homologous genes of *OsSPL9*, which have been proved to regulate tiller number and the grain yield of rice [52,58]. *PpSPL25* is homologous to *OsSPL15*, which has no published functional information. However, as shown in the phylogenetic tree, the high expression of *PpSPL25* in FM is consistent with the fact that it is homologous to *AtSPL14*, which plays essential roles in the transition from juvenile to adult. In conclusion, according to the homologous analysis, a total of 14 *PpSPLs* may participate in the reproductive process (*PpSPL2*/*5*/*6*/*8*/*14*/*16*/*18*/*23*/*26*) and flower organ development (*PpSPL5*/*13*/*14*/*15*/*16*/*17*/*21*/*26*/*27*). In particular, the 14 *PpSPLs* contain all eight significant, differentially expressed *PpSPLs* screened from transcriptome profiling, which indicates they are likely to play essential roles in flowering and reproductive growth in *P. pygmaeus*.

Many SPLs have been reported to be down-regulated by *miRNAs* through mRNA cleavage and/or translational repression [59]. In general, *miRNAs* can recognize miRNA responsive elements located in the coding regions, 5′ untranslated region (UTR), or 3′ UTR of the target SPLs. Specially, most SPLs have recognition sites complementary to *miR156* [60,61]. An adequate number of studies in many plant species, including Arabidopsis, rice, maize, and other plant species, confirm that the diverse regulatory functions of *miR156*-*SPLs* are conserved throughout the plant kingdom. *miR156*-*SPLs* modules play diverse functions in multiple developmental processes such as flowering time, reproductive organ development, leaf morphology, root development, etc., as well as response to various abiotic stresses, such as salt, drought, cold, and pathogen defense [22,33,45,48,52,54,61,62]. For example, more than 10 *AtSPLs*, including *AtSPL2*-*6*/*9*-*11*/*13*/*15*, were reported to be potential targets of *miR156*, which participate in developmental processes and abiotic stress responses [63,64]. In particular, *miR156*-*SPL*3/9 module illustrates a specific flowering regulation process in the age pathway in Arabidopsis [28,31,62]. In the study, we predicted 13 potential *miR156*-targeting SPLs from *P. pygmaeus*, including *PpSPL2*/*3*/*4*/*6*/*8*/*12*/*13*/*14*/*15*/*16*/*17*/*19*/*23*. Combined with transcriptome screening, four key *miR156*-targeting *PpSPLs*, including *PpSPL13*/*14*/*16*/*17*, were differentially expressed in the flowering and non-flowering *P. pygmaeus*, which are speculated to participate in flora transition and the flowering process in bamboo plants.

## 4. Materials and Methods

### 4.1. Plant Materials

*P. pygmaeus* plants were grown in the bamboo garden of Nanjing Forestry University, Jiangsu province (N 32°4′44″ N, E 118°48′17″). The dormant shoot buds (FE), germinated shoots (FM), and flower buds (FL) from flowering *P. pygmaeus* and the dormant shoot buds (NE), germinated shoots (NM), and leaf buds (NL) from non-flowering plants were harvested with six biological repeats, respectively. The 6 tissues with respective 3 biological repeats were rapidly frozen by liquid nitrogen and stored at −80 °C for RNA-Seq and RT-qPCR.

### 4.2. Library Preparation and Transcriptome Assembly

The 6 tissues with respective 3 biological repeats were sent to Novogene company (https://en.novogene.com/, Beijing, China) for RNA-Seq, accessed on 1 June 2020. RNA libraries were constructed by NEBNext^®^ Ultra™ RNA Library Prep Kit for Illumina (NEB, USA) with 1.5 μg RNA per sample. The clean sequencing data were obtained from raw reads by RSeQC. A de novo transcriptome assembly was performed by Trinity 2.2 and the transcript sequences were profiled by Corset. The raw sequencing data have been deposited in NCBI SRA with the accession number PRJNA648794. All the information can be referred from our previous study [35].

### 4.3. Identification and Sequence Analysis of SPL Family Genes from P. pygmaeu

We downloaded the Hidden Markov Model (HMM) profile of SBP domain from Pfam database (PF03110; http://pfam.janelia.org/search/sequence, Cambridge, Cambridgeshire, UK) and performed a local BLAST to screen *SPL* genes from the transcriptome of *P. pygmaeus*, accessed on 6 June 2022. We then used a set of high-quality settings with value < 1 × 10^−20^ to identify SPL genes based on SBP HMM. The presence of an intact SBP domain in candidate SPL genes were checked against Pfam (http://pfam.xfam.org/), SMART (http://smart.embl-heidelberg.de/, EMBL-Heidelberg, Germany), and NCBI Conserved Domains (http://www.ncbi.nlm.nih.gov/Structure/cdd/wrpsb.cgi, National Library of Medicine, Rockville Pike, Bethesda), accessed on 6 June 2022.

All SPL genes identified from *P. pygmaeus* were submitted to ExPASy ProtParam (https://web.expasy.org/protparam/, SIB Swiss Institute of Bioinformatics, Lausanne, Switzerland) for physicochemical property analysis and Wolf PSORT (https://wolfpsort.hgc.jp/, Tokyo, Japan) for subcellular localization prediction, accessed on 6 June 2022. MEME (http://meme-suite.org/tools/meme, National Institutes of Health, Rockville Pike, Bethesda, Maryland) was used to identify the conserved motifs of the SPL family, with repetition number setting as the multiple, maximum pattern number as 20, and optimal width as 6–200 residues, accessed on 7 June 2022.

### 4.4. Multiple Alignment and Phylogenetic Analysis of SPL Family Genes

The sequences of the other 7 plant species from SPL family members were downloaded from Phytozome v13 (https://phytozome.jgi.doe.gov/pz/portal.html, U.S. Department of Energy, Washington, USA), accessed on 8 June 2022. Multiple alignment was performed using MAFFT V7.407 with default parameters, which was then imported into GeneDoc software for visualization. The phylogenetic tree of the 183 SPL genes from 8 plant species was constructed using the ML method by RAxML(Random Axelerated Maximum Likelihood) with default parameters.

### 4.5. Calculation of Equivalent (Ks) and Non-Equivalent (Ka) Substitutions

The aligned sequences with the length of ≥300 bp and the identity of ≥40% were regarded as homologous pairs [65]. BLASTN was used to identify homologous SPL genes from *P. pygmaeus* and the other 7 plant species. Ks and Ka substitutions in each homologous pair were calculated as follows: (1) protein sequences of orthologous or paralogous pairs were aligned by MAFFT V7.407(RIMD, Osaka, Japan), accessed on 9 June 2022; (2) multiple aligned amino acid sequences were converted into nucleotide sequences using a homemade perl script based on original CDS sequences; (3) Ka and Ks substitutions were calculated using DnaSP5 software; (4) a sliding window analysis on Ka/Ks ratio was performed with a window parameter of 150 bp and step length of 9 bp.

### 4.6. MiRNA-Targeting PpSPLs Prediction

The potential targets of *miRNAs* were predicted by psRNATarget server (http://plantgrn.noble.org/psRNATarget, Noble Research Institute, USA) in all 28 *PpSPLs*, accessed on 9 June 2022. The relationship of *miRNAs* and their potential *PpSPL* targets was visualized by Cytoscape v3.3 (http://www.cytoscape.org/, National Resource for Network Biology, USA), accessed on 9 June 2022 [66].

### 4.7. Expression Pattern Analysis of PpSPLs by RNA-Seq

The expression pattern of 28 *PpSPL* genes was explored in the different tissues of flowering and non-flowering *P. pygmaeu* by RNA-Seq. In order to improve accuracy, we used CLCworkbench software (CLC Bio, Aarhus, Denmark) with full-length sequences of third-generation transcriptomes as a template to remap the clean data of next-generation transcriptomes and profile the mRNA abundance of each SPL gene, accessed on 10 June 2022. The heatmap of the 28 *PpSPLs* was drawn by R software (Lucent Technologies, Murray Hill, NJ, USA), accessed on 10 June 2022.

### 4.8. RT-qPCR Verification of PpSPLs

Total RNA of the 6 tissues with respective 3 biological repeats was extracted by Column Plant RNAout Kit (CAT#:71203, Tiandz, Beijing, China). The quantity of RNA was determined by Nanodrop 2000c, and the integrity of RNA was detected by Agilent 2100 and gel electrophoresis. The cDNA was synthesized using PrimerScript RT MasterMix (Takara, Tokyo, Japan). *Tubulin* (Genbank accession: gi|242385991) was used as a reference gene [35]. The specific primer pairs of 8 *PpSPLs* were designed using Primer Premier 5.0 (Supplemental Table S3). The relative expression level of the *PpSPLs* was calculated by the 2^−△△Ct^ method [67]. Mean values and deviations were calculated from three independent, biological experiments.

## 5. Conclusions

In the study, we identified and characterized a total of 28 SPLs from *P*. *pygmaeus* systematically. Based on RNA-Seq and RT-qPCR, the relative expression level of eight *PpSPLs* is significantly higher in flowering *P*. *pygmaeus* than that in non-flowering plants, including two highly expressed genes in FE (*PpSPL14* and *PpSPL16*), three in FM (*PpSPL21*, *PpSPL25* and *PpSPL27*), and three in FL (*PpSPL5*, *PpSPL13* and *PpSPL17*). Combined with phylogenetic analysis and homologous analysis, the eight significant, differentially expressed SPLs are likely to participate in the reproductive process and flower organ development. In addition, a total of 13 *PpSPLs* were predicted to have recognition sites complementary to *miRNA156*. Among them, four potential *miRNA156*-targeting *PpSPLs*, including *PpSPL13/14/16/17*, are highly expressed in flowering *P*. *pygmaeus*, which were speculated to participate in flora transition and the flowering process. Of significant interest to the study was the screening of 28 *PpSPLs* and the exploration of four key flowering related *PpSPLs* in *P*. *pygmaeus*, which fosters substantial insights into the underlying functions of SPLs in reproductive growth in bamboo plants.

## Data Availability

Not applicable.

## Supplementary Materials

The following supporting information can be downloaded at: https://www.mdpi.com/article/10.3390/ijms232214035/s1.
