# Supplementary material for: Genome-Wide Analysis of SQUAMOSA-Promoter-Binding Protein-like Family in Flowering Pleioblastus pygmaeus
Source: Int J Mol Sci. 2022 Nov 14;23(22):14035. doi: 10.3390/ijms232214035 (PMC9695801; doi:10.3390/ijms232214035)
Supplement: Supplementary file 1 [file ijms-23-14035-s001.zip › Supplemental Table S1.pdf]

Supplemental Table S1. Physicochemical properties of *SPL* family members in *Pleioblastus pygmaeus*

Name	AA	Molecular weight	Theoretical PI	Instability Index	Aliphatic Index	GRAVY	Rice Ortholog	Subcellular Location
PpSPL1	979	106995.9	5.35	51.38	80.17	-0.268	OsSPL6	nucleus
PpSPL2	448	48414.05	9.19	50.00	58.15	-0.576	OsSPL3	nucleus
PpSPL3	329	35159.27	8.78	67.67	55.99	-0.388	OsSPL19	nucleus
PpSPL4	370	39553.16	9.02	52.42	58.70	-0.481	OsSPL2	nucleus
PpSPL5	416	44078.82	8.46	62.13	65.58	-0.319	OsSPL5	nucleus
PpSPL6	463	50228.16	9.36	54.87	52.85	-0.649	OsSPL12	nucleus
PpSPL7	843	92946.38	5.66	53.42	80.06	-0.326	OsSPL9	nucleus
PpSPL8	464	50333.25	9.28	51.73	57.11	-0.572	OsSPL12	nucleus
PpSPL9	949	103439.99	5.85	53.98	78.10	-0.259	OsSPL6	nucleus
PpSPL10	567	62541.62	8.06	56.54	66.44	-0.550	OsSPL9	nucleus
PpSPL11	184	20144.74	9.75	72.36	54.78	-0.520	OsSPL19	nucleus
PpSPL12	400	42448.41	7.57	61.82	60.33	-0.422	OsSPL16	nucleus
PpSPL13	415	44347.85	7.13	59.72	65.37	-0.332	OsSPL18	nucleus
PpSPL14	383	39973.23	8.56	48.08	54.41	-0.518	OsSPL17	nucleus
PpSPL15	424	45024.57	8.21	55.99	65.61	-0.292	OsSPL18	nucleus
PpSPL16	381	39723.15	8.97	50.93	59.29	-0.426	OsSPL17	nucleus
PpSPL17	389	41255.68	10.83	62.04	68.53	-0.478	OsSPL7	nucleus
PpSPL18	308	33922.14	9.38	49.27	54.74	-0.587	OsSPL3	nucleus
PpSPL19	366	39517.98	8.53	50.12	59.56	-0.545	OsSPL2	nucleus
PpSPL20	875	97381.59	6.42	54.73	81.70	-0.323	OsSPL1	nucleus
PpSPL21	360	41021.97	8.15	58.50	80.97	-0.430	OsSPL9	nucleus
PpSPL22	587	64786.33	5.80	57.09	75.30	-0.420	OsSPL9	nucleus
PpSPL23	513	55999.47	8.52	56.90	56.69	-0.553	OsSPL3	nucleus
PpSPL24	272	29489.13	9.73	51.66	53.35	-0.659	OsSPL12	nucleus
PpSPL25	365	39236.18	9.52	65.24	76.14	-0.475	OsSPL15	nucleus
PpSPL26	154	17009.18	9.82	66.86	47.60	-0.871	OsSPL4	nucleus
PpSPL27	523	57339.02	5.66	56.83	75.39	-0.415	OsSPL9	nucleus
PpSPL28	1117	122036.59	7.17	55.05	79.12	-0.433	OsSPL15	nucleus
