# Supplementary material for: Genome-Wide Analysis of SQUAMOSA-Promoter-Binding Protein-like Family in Flowering Pleioblastus pygmaeus
Source: Int J Mol Sci. 2022 Nov 14;23(22):14035. doi: 10.3390/ijms232214035 (PMC9695801; doi:10.3390/ijms232214035)
Supplement: Supplementary file 1 [file ijms-23-14035-s001.zip › Supplemental Table S2.pdf]

Supplemental Table S2. Ka, Ks and Ka/Ks values of orthologous *SPL* pairs from *Oryza sativa*, *Phyllostachys edulis* and *Pleioblastus pygmaeus*

Gene pairs	Ka	Ks	Ka/Ks
OsSPL10/PhSPL11	0.189115	0.429814	0.439993
OsSPL10/PhSPL22	0.310584	0.589351	0.526992
OsSPL10/PhSPL28	0.337446	0.581801	0.580002
OsSPL10/PhSPL7	0.178123	0.462982	0.384729
OsSPL11/PhSPL10	0.19817	0.361607	0.548024
OsSPL11/PhSPL23	0.289396	0.637511	0.453947
OsSPL11/PhSPL6	0.220306	0.402241	0.547698
OsSPL12/PhSPL5	0.124281	0.461611	0.269234
OsSPL12/PhSPL9	0.120461	0.470187	0.256199
OsSPL13/PhSPL13	0.148304	0.376684	0.393708
OsSPL14/PhSPL17	0.133094	0.49812	0.267193
OsSPL14/PhSPL2	0.198941	0.73791	0.2696
OsSPL14/PhSPL25	0.216985	0.776747	0.279352
OsSPL14/PhSPL32	0.131273	0.458091	0.286565
OsSPL15/PhSPL15	0.078459	0.394493	0.198885
OsSPL15/PhSPL31	0.070986	0.39816	0.178286
OsSPL16/PhSPL14	0.157409	0.658266	0.239126
OsSPL16/PhSPL26	0.198221	0.926001	0.214061
OsSPL16/PhSPL3	0.192471	0.888647	0.216589
OsSPL16/PhSPL33	0.142452	0.639965	0.222593
OsSPL17/PhSPL17	0.193229	0.562141	0.343738
OsSPL17/PhSPL2	0.143193	0.500009	0.286381
OsSPL17/PhSPL25	0.150747	0.366073	0.411796
OsSPL17/PhSPL32	0.190733	0.593586	0.321323
OsSPL18/PhSPL26	0.128262	0.478881	0.267836
OsSPL18/PhSPL3	0.127651	0.492258	0.259317
OsSPL1/PhSPL19	0.129339	0.427635	0.302452
OsSPL2/PhSPL14	0.520758	1.092358	0.476729
OsSPL2/PhSPL18	0.102029	0.444038	0.229775
OsSPL2/PhSPL21	0.129179	0.451001	0.286428
OsSPL3/PhSPL24	0.150172	0.382302	0.392809
OsSPL3/PhSPL27	0.137817	0.357067	0.38597
OsSPL3/PhSPL5	0.236836	0.698734	0.338951
OsSPL3/PhSPL9	0.23413	0.736227	0.318014
OsSPL4/PhSPL10	0.245127	0.705713	0.347346

Gene pairs	Ka	Ks	Ka/Ks
OsSPL4/PhSPL23	0.185356	0.478486	0.38738
OsSPL4/PhSPL29	0.156228	0.545566	0.286359
OsSPL4/PhSPL6	0.264047	0.804568	0.328185
OsSPL5/PhSPL22	0.20395	0.455852	0.447404
OsSPL5/PhSPL28	0.227532	0.471747	0.482318
OsSPL6/PhSPL20	0.083561	0.349112	0.239352
OsSPL6/PhSPL30	0.0851	0.360257	0.236221
OsSPL7/PhSPL34	0.191434	0.398326	0.480595
OsSPL8/PhSPL35	0.067842	0.494638	0.137154
OsSPL9/PhSPL12	0.119254	0.428499	0.278306
OsSPL9/PhSPL8	0.096709	0.400207	0.241648
PpSPL10/OsSPL9	0.142974	0.517649	0.276199
PpSPL12/OsSPL16	0.162088	0.710093	0.228264
PpSPL12/OsSPL18	0.184215	0.826997	0.222752
PpSPL12/OsSPL2	0.528759	0.948517	0.557458
PpSPL13/OsSPL16	0.193584	0.927805	0.208648
PpSPL13/OsSPL18	0.122613	0.4813	0.254754
PpSPL14/OsSPL14	0.302285	0.909865	0.332231
PpSPL14/OsSPL17	0.229664	0.476703	0.481777
PpSPL15/OsSPL16	0.191222	0.872196	0.219242
PpSPL15/OsSPL18	0.126528	0.5028	0.251647
PpSPL16/OsSPL14	0.215419	0.772302	0.278931
PpSPL16/OsSPL17	0.143546	0.47328	0.303301
PpSPL17/OsSPL7	0.261931	0.470729	0.556437
PpSPL18/OsSPL3	0.175649	0.491868	0.357106
PpSPL19/OsSPL2	0.123629	0.449843	0.274826
PpSPL1/OsSPL6	0.088038	0.359624	0.244806
PpSPL20/OsSPL1	0.132407	0.438966	0.301634
PpSPL21/OsSPL9	0.078819	0.428807	0.183811
PpSPL22/OsSPL9	0.116519	0.490255	0.23767
PpSPL23/OsSPL3	0.147463	0.404496	0.364559
PpSPL25/OsSPL15	0.151034	0.526516	0.286855
PpSPL26/OsSPL4	0.282957	0.893586	0.316653
PpSPL27/OsSPL9	0.093675	0.445328	0.21035
PpSPL28/OsSPL15	0.072001	0.39616	0.181746
PpSPL2/OsSPL3	0.135902	0.366335	0.370978
PpSPL4/OsSPL2	0.119057	0.46719	0.254836

Gene pairs	Ka	Ks	Ka/Ks
PpSPL5/OsSPL10	0.316753	0.590254	0.536637
PpSPL5/OsSPL5	0.217194	0.489321	0.443867
PpSPL6/OsSPL12	0.125319	0.481129	0.260469
PpSPL7/OsSPL9	0.095892	0.382904	0.250432
PpSPL8/OsSPL12	0.112472	0.457174	0.246015
PpSPL9/OsSPL6	0.084111	0.375258	0.224142
PpSPL10/PhSPL12	0.07039	0.147603	0.476886
PpSPL10/PhSPL8	0.097133	0.253632	0.382967
PpSPL11/PhSPL1	0.090354	0.332282	0.27192
PpSPL11/PhSPL16	0.077678	0.123594	0.628492
PpSPL12/PhSPL14	0.027742	0.116722	0.237673
PpSPL12/PhSPL26	0.14103	0.627896	0.224606
PpSPL12/PhSPL3	0.1381	0.693772	0.199057
PpSPL12/PhSPL33	0.006633	0.017357	0.38215
PpSPL13/PhSPL14	0.148453	0.669777	0.221645
PpSPL13/PhSPL21	0.461623	1.142068	0.404199
PpSPL13/PhSPL26	0.005328	0.033732	0.157939
PpSPL13/PhSPL3	0.030884	0.215463	0.14334
PpSPL13/PhSPL33	0.133355	0.643041	0.207381
PpSPL14/PhSPL17	0.269884	0.641128	0.420951
PpSPL14/PhSPL2	0.158638	0.294494	0.538679
PpSPL14/PhSPL25	0.121865	0.164005	0.743055
PpSPL14/PhSPL32	0.277	0.606324	0.456852
PpSPL15/PhSPL14	0.158304	0.684572	0.231245
PpSPL15/PhSPL21	0.440904	1.161871	0.379478
PpSPL15/PhSPL26	0.032139	0.157674	0.203833
PpSPL15/PhSPL3	0.009551	0.023028	0.414749
PpSPL15/PhSPL33	0.123589	0.636257	0.194243
PpSPL16/PhSPL17	0.174118	0.565439	0.307935
PpSPL16/PhSPL2	0.011846	0.040327	0.293752
PpSPL16/PhSPL25	0.050949	0.141969	0.358876
PpSPL16/PhSPL32	0.181521	0.529748	0.342655
PpSPL17/PhSPL34	0.196159	0.380376	0.515697
PpSPL17/PhSPL37	0.165992	0.269549	0.615813
PpSPL18/PhSPL24	0.109172	0.22937	0.475964
PpSPL18/PhSPL27	0.076365	0.108009	0.707028
PpSPL18/PhSPL5	0.216495	0.778206	0.278198

Gene pairs	Ka	Ks	Ka/Ks
PpSPL18/PhSPL9	0.198514	0.75695	0.262255
PpSPL19/PhSPL18	0.042098	0.206038	0.204319
PpSPL19/PhSPL21	0.013326	0.046677	0.285483
PpSPL1/PhSPL20	0.013991	0.03265	0.428517
PpSPL1/PhSPL30	0.043537	0.151338	0.28768
PpSPL20/PhSPL19	0.0127	0.021128	0.60107
PpSPL21/PhSPL12	0.049435	0.156697	0.31548
PpSPL21/PhSPL8	0.037481	0.094675	0.395893
PpSPL22/PhSPL12	0.038689	0.082736	0.467621
PpSPL22/PhSPL8	0.069638	0.202818	0.343351
PpSPL23/PhSPL24	0.009295	0.02038	0.45607
PpSPL23/PhSPL27	0.048639	0.168474	0.288701
PpSPL23/PhSPL5	0.201802	0.637444	0.316579
PpSPL23/PhSPL9	0.189386	0.644778	0.293722
PpSPL24/PhSPL24	0.220794	0.70295	0.314096
PpSPL24/PhSPL27	0.22605	0.721958	0.313107
PpSPL24/PhSPL5	0.059336	0.120193	0.49367
PpSPL24/PhSPL9	0.074727	0.178133	0.419504
PpSPL25/PhSPL15	0.091276	0.125924	0.724852
PpSPL25/PhSPL31	0.093767	0.253718	0.369572
PpSPL26/PhSPL23	0.19434	0.482431	0.402835
PpSPL26/PhSPL29	0.1945	0.192802	1.008803
PpSPL26/PhSPL6	0.272237	1.023698	0.265935
PpSPL27/PhSPL12	0.06322	0.205168	0.308139
PpSPL27/PhSPL8	0.036854	0.067406	0.546742
PpSPL28/PhSPL15	0.035173	0.118265	0.297406
PpSPL28/PhSPL31	0.008096	0.031066	0.260593
PpSPL2/PhSPL24	0.05187	0.172549	0.300612
PpSPL2/PhSPL27	0.011691	0.033018	0.354095
PpSPL2/PhSPL5	0.181297	0.638563	0.283914
PpSPL2/PhSPL9	0.178062	0.622518	0.286036
PpSPL3/PhSPL1	0.049257	0.216106	0.227928
PpSPL3/PhSPL16	0.061571	0.104837	0.587299
PpSPL4/PhSPL18	0.011133	0.0342	0.325531
PpSPL4/PhSPL21	0.042701	0.155621	0.274389
PpSPL4/PhSPL3	0.490928	0.986482	0.497655
PpSPL5/PhSPL11	0.25113	0.55881	0.449401

Gene pairs	Ka	Ks	Ka/Ks
PpSPL5/PhSPL22	0.009046	0.039097	0.231374
PpSPL5/PhSPL28	0.064902	0.113903	0.569805
PpSPL5/PhSPL7	0.278913	0.615453	0.453182
PpSPL6/PhSPL24	0.197297	0.59229	0.333109
PpSPL6/PhSPL27	0.186588	0.579214	0.322139
PpSPL6/PhSPL5	0.045223	0.12313	0.367278
PpSPL6/PhSPL9	0.005608	0.029114	0.192634
PpSPL7/PhSPL12	0.043297	0.156947	0.27587
PpSPL7/PhSPL8	0.010898	0.029484	0.369628
PpSPL8/PhSPL24	0.195233	0.647657	0.301445
PpSPL8/PhSPL27	0.184119	0.619999	0.296966
PpSPL8/PhSPL5	0.017131	0.023368	0.733092
PpSPL8/PhSPL9	0.035436	0.115744	0.306156
PpSPL9/PhSPL20	0.040716	0.149962	0.271511
PpSPL9/PhSPL30	0.010663	0.0291	0.366429
