# Supplementary material for: Genome-Wide Analysis of SQUAMOSA-Promoter-Binding Protein-like Family in Flowering Pleioblastus pygmaeus
Source: Int J Mol Sci. 2022 Nov 14;23(22):14035. doi: 10.3390/ijms232214035 (PMC9695801; doi:10.3390/ijms232214035)
Supplement: Supplementary file 1 [file ijms-23-14035-s001.zip › Supplemental Table S3.pdf]

Supplemental Table S3. Primer list of reference gene and *PpSPLs* in *Pleioblastus pygmaeus*

Gene Abbreviation	Forward primers	Reverse primers	Rice orthologlocus
TUB	TGACATTGAGCGCCCAACTTACA	ATCCACATTCAGAGCACCATCGA	Os03g0726100
PpSPL5	GCGAGGAGCAGCAGCACTTC	GATACCGACGAGCACGACAAGATG	NM_001409348
PpSPL13	GTCCAGCCAACCGAGCACATC	GGGAGCAAGCAAACCAGGAAGAG	XM_015755388
PpSPL14	GCAACTACCTGGCGGCAAGC	CGTGATGAACTGAGCCTGGATGG	XM_015755476
			XM_006660710
PpSPL16	CAGTTCACCACGGTCAGTTCTCAG	GCTCGACTTGCGGCGGATTC	XM_015755476
			XM_006660710
PpSPL17	GGGCTCGCTTCGCTTTGCTG	CGGCTGCTGCTGCTGTAAGTAC	XM_015779858
PpSPL21	ACACGCTGCTGCTGTCATGC	TGTGCCGCTCCAACTTTCTTCTAC	XM_015784566
PpSPL25	CTTCCGTGGCTTCTTCACCTTCTC	GGTAGCTTCCGCATCCGTTTCC	XM_015794435
PpSPL27	ACACGCTGCTGCTGTCATGC	TGTGCCGCTCCAACTTTCTTCTAC	XM_015784566
